# Editorial: Technological strategies to improve animal health and production

**DOI:** 10.3389/fvets.2023.1206170

**Published:** 2023-05-24

**Authors:** Daniel Hernandez-Patlan, Guillermo Tellez-Isaias, Xochitl Hernandez-Velasco, Bruno Solis-Cruz

**Affiliations:** ^1^Laboratorio 5: Laboratorio de Ensayos de Desarrollo Farmacéutico (LEDEFAR), Unidad de Investigacion Multidisciplinaria, Facultad de Estudios Superiores (FES) Cuautitlan, Universidad Nacional Autonoma de Mexico, Cuautitlán Izcalli, Mexico; ^2^División de Ingeniería en Nanotecnología, Universidad Politécnica del Valle de México, Tultitlan, Mexico; ^3^Department of Poultry Science, University of Arkansas, Fayetteville, AR, United States; ^4^Departamento de Medicina y Zootecnia de Aves, Facultad de Medicina Veterinaria y Zootecnia, Universidad Nacional Autonoma de Mexico, Ciudad de México, Mexico

**Keywords:** animal health, animal production, genetic improvement, nutritional strategies, technological strategies

## Introduction

As animal health and production are crucial factors in the agricultural industry, the health of livestock directly affects their productivity and, consequently, the income of farmers. Technological advances have contributed to animal health and production practices in recent years. This Research Topic focused on using promising technological techniques to reduce animal illness incidence and significance to improve animal health and output. This was achieved through the collection of 25 original research articles, 5 review articles, 3 systematic review articles and one method article, it brings together 212 leading authors in cutting-edge research issues on technology for diagnosing, preventing, controlling, and treating animal diseases important to public health and animal production.

The livestock sector expansion and change offer significant prospects for agricultural development, poverty reduction, food security, and human nutrition. Large-scale livestock production and food chains meet the expanding demand for animal products in many nations. Food safety and public health depend on efficient, healthy, and safe livestock production. It is therefore that several techniques to improve and increase the current agricultural production of the different animal species are being developed and evaluated, including drug delivery systems such as micro/nano systems; disease management through the use of vaccines, alternatives to antibiotics, or nutraceuticals; improvement of animal nutrition with prebiotics, probiotics, biosorbents, or bioactive substances; reproductive technologies; precision livestock farming and even employing genetic improvements.

## Genetic improvement

Genetic enhancement improves animal health and output. Since traditional methods are not always effective, genetic selection and breeding programs have been developed to enhance desirable traits in animals, such as increased milk production or disease resistance (1, 2). Through selective breeding, farmers can improve the genetic traits of their livestock and enhance their productivity. In addition, molecular genetics has made it possible to identify specific genes responsible for desirable traits, allowing for more targeted breeding programs. Genome editing technologies allow programmable DNA nucleases-based accurate genome alterations (3). Despite its many benefits, genetic improvement in livestock creates technological, ethical, and societal issues (4–7); hence this technology has not yet completely replaced the rest of the technological options that have been used for the purpose of improving animal health and production.

Most genetic technologies are related to the direct manipulation of DNA oriented to the expression of certain genes. Nonetheless, there are many other applications based on the analysis of DNA information aimed at improving livestock performance, within which bioinformatics tools for data analysis, correlation and extraction can be considered. For instance, weighted gene co-expression network analysis (WGCNA) is employed to recognize the functional relationships between genes and identify, through hierarchical clustering, groups of genes with correlated expression that may be involved in the pathogenesis of subacute ruminal acidosis (SARA) (Wang Q. et al.). This study identified hub genes to explain SARA's molecular biological and metabolic processes, suggesting ways to lower SARA risk in the future.

On the other hand, another technological tool based on the use or manipulation of nucleic acids is the use of microRNAs (miRNAs) as micro-regulators of gene expression at the post-transcriptional level in various cell types and physiological processes. Liver lipid metabolism and homeostasis require miRNAs. Their involvement in fatty liver syndrome (FLS) etiology is uncertain. Since FSL is a prevalent metabolic in laying hens, and no specific therapeutic techniques have been established, miRNA expression profiles and processes have been investigated (Zhu et al.). This study showed that miR-216/miR-217 cluster, is implicated in several metabolic pathways, supporting the association between miRNA expression levels and FLS in laying chickens. These findings shed light on miRNAs' functions in FLS pathogenesis in laying hens, which could lead to new treatment options.

## Nutritional strategies

In recent years, nutritional strategies undoubtedly have received the greatest boom and attention. Since adequate nutrition is essential for the proper growth and development of animals, it is important to provide animals with a balanced diet that meets their specific nutritional needs. Probiotics and prebiotics in feed promote digestion and animal health. Antibiotics used to combat gastrointestinal infections and minimize gut stresses promoted growth (8). This strategy to improve livestock production, even if used at a subtherapeutic level of antibiotics, had an important role in enhancing livestock production for many years. However, since this practice led to a sanitary emergency associated with antibiotic resistance of animals and important human pathogens, it has been globally discontinued (9, 10).

This situation has forced the livestock industry, and researchers from around the world have focused their attention on developing substantial improvements in livestock productivity, particularly the improvement of nutritional strategies that could modify animal metabolism in specific and direct ways, such as reduction in systemic inflammation, and chronic stress, which is reflected in the enhancement of production efficiency; improvement of carcass composition in growing animals; increasing milk production in lactating animals; and reducing animal waste per unit of production, but also with the livestock health and welfare (11, 12).

To minimize the detrimental consequences of removing antibiotic growth boosters from animal diets, various animal nutrition techniques have been devised and tested. Nutrition impacts host defense and disease resistance. Among the most common animal feed additives are probiotics, prebiotics, enzymes, bacteriocins, essential oils, herbs, spices, phytogenic compounds, minerals, and organic acids. Each has a beneficial effect through different biological mechanisms (13–16).

In this Research Topic, Malik et al. utilized a meta-analysis of a comprehensive review to assess the effects of dietary Cr supplementation on dry matter intake (DMI), and milk output. This mineral improves dairy cow performance, but most feed ingredients for cows have low Cr concentrations. The meta-analysis of previously published literature indicated that Cr supplementation raises DMI and is less effective before parturition. This study demonstrated that Cr supplement enhances milk production in multiparous and primiparous cows. Finally, Cr supplementation did not affect milk protein, fat, lactose, or solids-not-fat.

A vitamin A meta-analysis conducted by Li W. et al. to better comprehend vitamin A and intramuscular fat levels and provide clues for future research and commercial use. Cattle need fat-soluble vitamin A for vital functions including vision. The meta-analysis indicated vitamin A may reduce intramuscular fat in bovine steers.

Ma et al. studied alfalfa supplementation as pig feed ingredient to alleviate various problems in the pig industry and to improve pig production performance, describing remarkable benefits in growth and reproductive performance, meat quality, and intestinal health.

Probiotic feed supplements are another popular way to improve livestock operations since they reduce disease and boost animal performance. Probiotic dietary additives in animal feeds have been successful for many genus and bacterial strains. Probiotics can treat many illnesses and ailments.

Necrotic enteritis (NE), caused by *Clostridium perfringens*, threatens broilers' intestinal health. It's poultry's worst disease because it slows growth and costs money (17, 18). Li P. et al. examined the effects of feeding *Lactobacillus fermentum* (LF) and *Lactobacillus paracasei* (LP) on intestinal health and growth performance of broilers challenged with coccidia and *C. perfringens* (CCP) in NE model reducing the severity of NE.

Zou et al. studied how a probiotic complex (PC) comprising *Bacillus subtilis, Clostridium butyricum*, and *Enterococcus faecalis* affected AA+ male broiler productivity, carcass characteristics, immune organ indices, fecal microbiota counts, and noxious gas emissions. Probiotic complexes improved immune organ development, reduce *Escherichia coli* and *Salmonella* in feces, increase *Lactobacillus*, and reduce NH_3_ and H_2_S emissions, supporting their use in broilers.

Other combinations of one or more species of probiotic bacteria and prebiotic ingredients, known as synbiotics. A multicomponent synbiotic with amylase, cellulase, xylanase, β-gluconate, protease, phytase, live probiotic cultures (*B. subtilis* and *Bacillus licheniformis*), mannan oligosaccharide prebiotic culture, and silicon dioxide was tested by Trukhachev et al.. These studies investigated whether this this combined additive (probiotic, prebiotic, and enzymes) could improve rumen fermentation. The synbiotic supplement did not affect early lactation milk production in highly productive Russian Holstein lactating cows. However, it improved rumen fermentation, improving feed consumption without affecting blood characteristics. It also doesn't harm supplemented animals.

Chen et al. tested a simpler lactic acid bacteria mixture (*L. plantarum* PS-8, *L. plantarum* PS-F, and L. buchneri HM-01) and molasses. They found that this combination increased rice straw digestibility and rumen microbial colonization, making it an ideal pretreatment and an alternative method for improving rice straw quality.

However, the use of probiotic microorganisms is one of many nutritional strategies that has been evaluated as a strategy to improve livestock production. Using plant products, their metabolites, essential oils, or extracts has also been a very commonly evaluated strategy. In this Research Topic, many natural products were determined as natural feed supplements, showing promising results. Hassanein et al. tested sun-dried Azolla (*Azolla pinnata*) meal protein in concentrated feed combinations to substitute sunflower meal protein in nursing Zaraibi goats. This replacement improved nutritional digestibility, milk output, composition, and economic feed efficiency.

Mango seeds (MS) also were tested to investigate the effect of replacing yellow corn grain with them (El-Sanafawy et al.). The results demonstrated that replacing maize grain with MS increased digestibility, milk output, feed conversion, and economic efficiency without affecting Damascus goat performance, providing a novel energy source for this livestock species.

Yang et al. examined the effects of tea tree oil (TTO)-supplemented diets on finishing pigs' growth, meat quality, serum biochemical indices, and antioxidant capacity showing improved growth performance, meat quality, serum biochemical indices, and antioxidant capacity in finishing pigs by modulating the expression of genes associated with meat quality and intramuscular fat content.

Jiang et al. examined the effects of replacing isonitrogenous and isoenergetic alfalfa hay (AH) with stevia (*Stevia rebaudiana*) hay (SH) in dairy cow diets. This study examined how substituting stevia hay (SH) for AH in dairy cow diets impacts nutritional digestion, lactation performance, nitrogen use, and rumen fermentation demonstrating that SH can partially replace AH in dairy cow diets on an isonitrogenous and isoenergetic basis without affecting intake, increasing milk output, milk fat content, nutritional digestibility, and nitrogen utilization.

Many other options, based on the nutritional supplementation of animals with a great variety of substances of different chemical nature, have also been evaluated. This is extremely important for the poultry production system since feed contains essential nutrients for growth and health which also help to improve the quality of meat and eggs. For example, the effect of dietary supplementation with N-Carbamylglutamate, an activator for endogenous arginine synthesis, has been tested on production performance, egg quality, and uterine gene expression in layers (Ma et al.). Tannins of different sources have also been evaluated as nutritional strategies to examinate their influence on immune function and liver health of broiler chickens (Yunan et al.), as well as to test their ability to control pathogens and regulate microbial nitrogen metabolism during poultry litter composting (Arzola-Alvarez et al.). 25-hydroxycholecalciferol (25OHD_3_), a vitamin D3 metabolite, has also been considered in poultry production for different purposes, such as the evaluation of its dietary inclusion on duodenal development and local intestinal innate immunity in young broiler chickens (Leiva et al.); but also to determine its impact when it is used over time in broilers feed as a modulator of the intestinal cytokine abundance and epithelial barrier integrity (Abascal-Ponciano et al.). On the other hand, the influence of different dietary protein sources and litter condition, two commonly used practices in an antibiotic-free broiler production, have also been evaluated on intestinal cell mitotic activity and local intestinal immunological responses (Keel et al.). Not only protein sources have been considered, but also some amino acids, such as L-arginine. The effect of the *in ovo* feeding of this amino acid was evaluated as a strategy to improve breast muscle weight, muscle morphology, amino acid profile, and gene expression of muscle development in slow-growing chickens (Lu et al.).

In addition to probiotics, prebiotics, essential oils, and enzymes used as nutritional strategies for ruminant livestock production, other options of a different chemical nature have also been considered as options to modify their digestion kinetics, leading to an improved production efficiency. In this Research Topic, the use of different methionine hydroxy analogs (MHA) in Hu sheep was explored as source of methionine on the rumen microbiota and metabolome, also determining their relationship (Li S. et al.). This study showed that nutritional supplementation with MHA significantly improved the richness and diversity of the ruminal microbiota and stimulated the ruminal fermentation by increasing concentrations of total VFA, acetate, and propionate. Bioactive substances such as flavonoids, phenols, and terpenes obtained from plants, have also shown to possess anti-inflammatory and bacteriostatic effects. *Astragalus membranaceus* (AM), a leguminous widely used as medicinal plant, was evaluated as a feed additive of dairy cows (Huang et al.). Results of this study shown that AM inclusion positively impacts the reproductive performance, immunity, and endocrine of dairy cows during the perinatal period, giving dairy farmers an alternative strategy to ensure the safe transition of cows during the perinatal period. Silage additives and silage pre-treatments also have been recommended as an innovative and viable strategy for the ruminant nutrition, that may preserve roughage and provide animals with a nutritious feed source. Herein, two different silage pre-treated with ferrous sulfate heptahydrate (FSH) were evaluated as an innovative strategy for ruminant nutrition. The first one consisted of a Black Cane (*Saccharum sinensis* R.) silage containing FSH, aimed to assess whether this pre-treatment might influence fermentation quality, anthocyanin stability, ruminal biogases, rumen fermentation profile, and the microbial populations of black cane silages (Suong et al.). Secondly, the effect of a standard total mixed ration containing pre-treated anthocyanin-rich black cane silage with FSH was investigated on animal performance, blood biochemical indices, rumen fermentation, microbial community, and carcass characteristics in meat goats (Purba et al.). The results of this study demonstrated that this nutritional strategy reduces oxidative stress and makes the meat more tender.

Finally, rabbit farming is one of the fastest growing micro-livestock enterprises, and it has become very important in developing countries because it can be considered as a source of healthy meat with high nutritional value (19, 20). In this Research Topic, a study carried out to examine the effects of yucca extract alone and in combination with *Clostridium butyricum* on growth performance, nutrient digestibility, muscle quality, and intestinal development of weaned rabbits, demonstrated that using this combination in feed had a synergistic effect on nutrient digestibility and improved growth performance (Wang Y. et al.). It is also presented a study aimed to evaluate the use of mealworm (*Tenebrio molitor*) frass (TMF) in rabbit diets, as well as its effect on growth performance, blood profiles, carcass characteristics, meat quality, and fatty acid profiles of rabbit meat (Radwan et al.). Authors found that TMF has the potential for use in the feeding system of rabbits without unfavorable effects on growth performance and carcass traits, as well as improving meat quality parameters.

## Disease management

Animal diseases have direct and indirect biophysical and socioeconomic effects that range from local to global (21). These illnesses cause animal welfare, productivity losses, food insecurity, economic loss, and health issues in cattle production systems. Disease control is another important technique for animal health and output. The development and use of diagnostic tools, vaccines, antibiotics, and other drugs can help prevent and treat animal diseases. Farmers can also implement animal husbandry practices and biosecurity measures to prevent the spread of disease on their farms (22, 23).

Mycotoxicosis illnesses are vital to animal health and production outside of the food chain. Mycotoxins reduce immunity, making people more susceptible to diseases, and vaccination failure costs animal businesses a lot of money. Thus, several methods have been developed to reduce cattle mycotoxin exposure.

Vázquez-Durán et al. reviewed the most important agro-waste-based materials that can adsorb aflatoxins showing promising results to reduce the adverse effects of aflatoxin B1.

*In-ovo* technology has been used to administer carbohydrates, amino acids, hormones, prebiotics, probiotics, synbiotics, antibodies, immunostimulants, minerals, and microorganisms with various physiological effects. The Kpodo and Proszkowiec-Weglarz review is an outstanding comprehensive review that describes several benefits of these supplements in broiler chickens.

As mentioned above, diagnostic technologies and methods are strategic tools for improving animal health and production. Thus, Ayala et al. performed a next-generation sequencing investigation to characterize the cecal, ileal, cloacal, and cutaneous microbiota of focal ulcerative dermatitis (FUDS) in laying hens to discover its causes through next generation sequencing (NGS). They isolated pathogens, found connections between isolates, and created a Direct Fed Microbial (DFM) combination that inhibits FUDS. *Staphylococcus aureus* and agents caused FUDS in laying hens, and six virulence factors related to adhesion, enzyme, immune evasion, secretion system, toxin, and iron uptake were found. *In vitro*, the *Bacillus pumilus*-based DFM suppressed both infections, reducing FUDS mortality and increasing egg production.

Animal disease control platforms use nanotechnological techniques. Nanotechnology has transformed commercial use of nano-sized materials in medical, food, biotechnology, pharmaceuticals, and more. Hence, Baholet et al. summarized the available data on zinc nanoparticles' effects on gastrointestinal microbiota and their impact on post-weaning diarrhea.

## Reproductive technologies

Reproductive technology has improved animal health and output during the last century. These technologies have transformed *in vitro* and *in vivo* livestock reproductive biology research. Artificial insemination and embryo transfer allow animals with desirable genetic features to be bred anywhere. *In vitro* fertilization and cloning can be used to produce animals with a specific trait to improve productivity, health, and production cycles to maximize herd performance parameters, especially for milk, meat, and replacement animals (24, 25).

## Precision livestock farming

Precision Livestock Farming (PLF) uses digital technologies to monitor and control livestock output. It uses process engineering to autonomously monitor, model, and manage livestock production (26). PLF can boost production, animal welfare, and environmental sustainability in livestock farming (27). Sensors can track animal behavior, health, and growth to identify issues early and improve animal health and output. PLF may also lower farm environmental effect by supporting management practices (28). Even though PLF has improved animal health and welfare, traceability, and livestock producer value, many of these technologies are still unproven, and only a few that focus on animal welfare have been commercialized and adopted (29).

One of the main requirements in the development of PLF has been the development and deployment of technologies that provide livestock producers with accurate and relevant information, for which digitalization will help to achieve these goals. Digital Livestock Farming Systems are currently commercialized as novel strategies using big data synergized with information and communication technology (ICT), Internet of Things (IoT), wireless biometric sensors, mobile phones, artificial intelligence (AI), autonomous systems, and drones for livestock producers, consumers, and farm animals (29–31).

PLF and Digital Livestock Farming Systems could also boost other animal health and productivity technologies. More information is needed to apply both technologies. Thus, a recent study examined how digital livestock systems and probiotic combinations could boost swine productivity in response to climate change. This study suggests that digital livestock systems could improve livestock production, reduce the livestock industry's environmental impact, save energy, and improve animal welfare, especially in the swine industry (Park and Seo).

## Conclusion

Technology can increase animal health and production. Genetic improvement, nutritional strategies, disease management, reproductive technologies, and precision livestock farming are all effective strategies that can be used alone or combined to enhance animal productivity. As technology advances, new strategies will likely be developed, further improving animal health and production.

## Author contributions

DH-P and BS-C: writing—original draft preparation. GT-I: reviewing the article. XH-V: formatting and editing. All authors have read and approved the submitted version.

## References

[1] Perisse IV Fan Z Singina GN White KL Polejaeva IA. Improvements in gene editing technology boost its applications in livestock. Front Genet. (2021) 11:614688. 10.3389/fgene.2020.614688 33603767 PMC7885404

[2] Wang S Qu Z Huang Q Zhang J Lin S Yang Y. Application of gene editing technology in resistance breeding of livestock. Life. (2022) 12:12. 10.3390/life12071070 35888158 PMC9325061

[3] Raza SHA Hassanin AA Pant SD Bing S Sitohy MZ Abdelnour SA. Potentials, prospects and applications of genome editing technologies in livestock production. Saudi J Biol Sci. (2022) 29:1928–35. 10.1016/j.sjbs.2021.11.037 35531207 PMC9072931

[4] Biscarini F Nicolazzi E Alessandra S Boettcher P Gandini G. Challenges and opportunities in genetic improvement of local livestock breeds. Front Genet. (2015) 5:1–16. 10.3389/fgene.2015.00033 25763010 PMC4340267

[5] Bruce A. Genome edited animals: learning from GM crops? Transgenic Res. (2017) 26:385–98. 10.1007/s11248-017-0017-2 28432545 PMC5422448

[6] Ishii T. Genome-edited livestock: ethics and social acceptance. Anim Front. (2017) 7:24–32. 10.2527/af.2017.0115

[7] Middelveld S Macnaghten P. Gene editing of livestock: sociotechnical imaginaries of scientists and breeding companies in the Netherlands. Elem Sci Anth. (2021) 9:00073. 10.1525/elementa.2020.00073

[8] Haulisah NA Hassan L Bejo SK Jajere SM Ahmad NI. High levels of antibiotic resistance in isolates from diseased livestock. Front Vet Sci. (2021) 8:652351. 10.3389/fvets.2021.652351 33869326 PMC8047425

[9] Cameron A Esiovwa R Connolly J Hursthouse A Henriquez F. Antimicrobial resistance as a global health threat: the need to learn lessons from the COVID-19 pandemic. Glob Policy. (2022) 13:179–92. 10.1111/1758-5899.13049 35601654 PMC9111155

[10] Dadgostar P. Antimicrobial resistance: implications and costs. Infect Drug Resist. (2019) 12:3903–10. 10.2147/idr.s234610 31908502 PMC6929930

[11] Rashid Z Mirani ZA Zehra S Gilani SMH Ashraf A Azhar A. Enhanced modulation of gut microbial dynamics affecting body weight in birds triggered by natural growth promoters administered in conventional feed. Saudi J Biol Sci. (2020) 27:2747–55. 10.1016/j.sjbs.2020.06.027 32994734 PMC7499368

[12] Valenzuela-Grijalva NV Pinelli-Saavedra A Muhlia-Almazan A Domínguez-Díaz D González-Ríos H. Dietary inclusion effects of phytochemicals as growth promoters in animal production. J Anim Sci Technol. (2017) 59:8. 10.1186/s40781-017-0133-9 28428891 PMC5392986

[13] Evangelista AG Corrêa JAF Pinto ACSM Luciano FB. The impact of essential oils on antibiotic use in animal production regarding antimicrobial resistance–a review. Crit Rev Food Sci Nutr. (2022) 62:5267–83. 10.1080/10408398.2021.1883548 33554635

[14] Kholif AE Anele UY Patra AK Varadyova Z. Editorial: The use of phytogenic feed additives to enhance productivity and health in ruminants. Front Vet Sci. (2021) 8:685262. 10.3389/fvets.2021.685262 34017872 PMC8129181

[15] Liu Y Espinosa CD Abelilla JJ Casas GA Lagos LV Lee SA. Non-antibiotic feed additives in diets for pigs: a review. Anim Nutr. (2018) 4:113–25. 10.1016/j.aninu.2018.01.007 30140751 PMC6103469

[16] Stevanović ZD Bošnjak-Neumüller J Pajić-Lijaković I Raj J Vasiljević M. Essential oils as feed additives—future perspectives. Molecules. (2018) 23:1717. 10.3390/molecules23071717 30011894 PMC6100314

[17] Skinner JT Bauer S Young V Pauling G Wilson J. An economic analysis of the impact of subclinical (mild) necrotic enteritis in broiler chickens. Avian Dis. (2010) 54:1237–40. 10.1637/9399-052110-Reg.1 21313845

[18] Timbermont L Haesebrouck F Ducatelle R Van Immerseel F. Necrotic enteritis in broilers: an updated review on the pathogenesis. Avian Pathol. (2011) 40:341–7. 10.1080/03079457.2011.590967 21812711

[19] Krupová Z Wolfová M Krupa E Volek Z. Economic values of rabbit traits in different production systems. Animal. (2020) 14:1943–51. 10.1017/S1751731120000683 32264994

[20] Mutsami C Karl S. Commercial rabbit farming and poverty in urban and peri-urban Kenya. Front Vet Sci. (2020) 7:353. 10.3389/fvets.2020.00353 32656255 PMC7325956

[21] Thornton PK. Livestock production: recent trends, future prospects. Philos Trans R Soc Lond Biol Sci. (2010) 365:2853–67. 10.1098/rstb.2010.0134 20713389 PMC2935116

[22] Bate AM Jones G Kleczkowski A Naylor R Timmis J White PCL. Livestock disease management for trading across different regulatory regimes. Ecohealth. (2018) 15:302–16. 10.1007/s10393-018-1312-y 29435773 PMC6132418

[23] Derner JD Hunt L Filho KE Ritten J Capper J Han G. Livestock production systems. In:Briske DD, editor. Rangeland Systems, Processes, Management and Challenges. Springer Series on Environmental Management. Cham: Springer (2017), p. 347–72. 10.1007/978-3-319-46709-2

[24] Aguila L Osycka-Salut C Treulen F Felmer R. Pluripotent core in bovine embryos: a review. Animals. (2022) 12:1010. 10.3390/ani12081010 35454256 PMC9032358

[25] Landeo L Zuñiga M Gastelu T Artica M Ruiz J Silva M. Oocyte quality, in vitro fertilization and embryo development of alpaca oocytes collected by ultrasound-guided follicular aspiration or from slaughterhouse ovaries. Animals. (2022) 12:1102. 10.3390/ani12091102 35565530 PMC9102040

[26] Tullo E Finzi A Guarino M. Review: Environmental impact of livestock farming and precision livestock farming as a mitigation strategy. Sci Total Environ. (2019) 650:2751–60. 10.1016/j.scitotenv.2018.10.018 30373053

[27] Schillings J Bennett R Rose DC. Exploring the potential of precision livestock farming technologies to help address farm animal welfare. Front Vet Sci. (2021) 2:639678. 10.3389/fanim.2021.639678

[28] Bigliardi B Bottani E Casella G Filippelli S Petroni A Pini B. Industry 40 in the agrifood supply chain: a review. Procedia Comput Sci. (2023) 217:1755–64. 10.1016/j.procs.2022.12.375

[29] Tuyttens FAM Molento CFM Benaissa S. Twelve threats of precision livestock farming (PLF) for animal welfare. Front Vet Sci. (2022) 9:889623. 10.3389/fvets.2022.889623 35692299 PMC9186058

[30] da Rosa Righi R Goldschmidt G Kunst R Deon C André da Costa C. Towards combining data prediction and internet of things to manage milk production on dairy cows. Comput Electron Agric. (2020) 169:105156. 10.1016/j.compag.2019.105156

[31] Wolfert S Ge L Verdouw C Bogaardt MJ. Big data in smart farming – a review. Agric Syst. (2017) 153:69–80. 10.1016/j.agsy.2017.01.023

